# The NLRP3-Caspase 1 Inflammasome Negatively Regulates Autophagy via TLR4-TRIF in Prion Peptide-Infected Microglia

**DOI:** 10.3389/fnagi.2018.00116

**Published:** 2018-04-18

**Authors:** Mengyu Lai, Hao Yao, Syed Zahid Ali Shah, Wei Wu, Di Wang, Ying Zhao, Lu Wang, Xiangmei Zhou, Deming Zhao, Lifeng Yang

**Affiliations:** National Animal Transmissible Spongiform Encephalopathy Laboratory, College of Veterinary Medicine, State Key Laboratories for Agrobiotechnology, Key Laboratory of Animal Epidemiology and Zoonosis, Ministry of Agriculture, China Agricultural University, Beijing, China

**Keywords:** prion diseases, PrP106-126, NALP3 inflammasome, autophagy, microglia

## Abstract

Prion diseases are neurodegenerative disorders characterized by the accumulation of misfolded prion protein, spongiform changes in the brain, and brain inflammation as a result of the wide-spread activation of microglia. Autophagy is a highly conserved catabolic process for the clearance of cytoplasmic components, including protein aggregates and damaged organelles; this process also eliminates pathological PrP^Sc^ as it accumulates during prion infection. The NALP3 inflammasome is a multiprotein complex that is a component of the innate immune system and is responsible for the release of pro-inflammatory cytokines. Our previous study showed that the neurotoxic prion peptide PrP106-126 induces NALP3 inflammasome activation and subsequent IL-1β release in microglia. Autophagy is involved in the regulation of the immune responses and inflammation in many diseases including neurodegenerative diseases. However, the relationship between autophagy and NALP3 inflammasome in prion diseases has not been investigated. In this study, we demonstrated that the processing and release of mature IL-1β is significantly enhanced by the inhibition of autophagy. Conversely, gene-silencing of the NALP3 inflammasome promotes autophagy. Suppression of TRIF or TLR4 by siRNA attenuated PrP106-126-induced autophagy, which is indicating that the TLR4-TRIF signaling pathway is involved in PrP106-26-induced autophagy. Caspase 1 directly cleaved TRIF to diminish TLR-4-TRIF mediated autophagy. Our findings suggest that the inhibition of autophagy by NALP3 inflammasome is probably mediated by activated Caspase-1-induced TRIF cleavage. This is the first study reporting that the NALP3 inflammasome complex negatively regulates autophagy in response to PrP106-126 stimulation in microglia, and partly explains the mechanism of autophagy inhibition by Caspase-1 in PrP106-126-induced BV2 cell activation. Our findings suggest that autophagy up-regulation and inhibition of Caspase-1 may protect against prion-induced neuroinflammation and accelerate misfolded protein degradation and are potential therapeutic approaches for prion diseases.

## Introduction

Prion diseases, also known as transmissible spongiform encephalopathies (TSEs), are a family of fatal progressive neurodegenerative disorders that affect humans and several species of animals. They are characterized by extensive spongiform degeneration, widespread neuronal loss, synaptic alterations, atypical brain inflammation, and microgliosis (Soto and Satani, 2011). The causative agents of TSEs are believed to be prions, which are able to induce abnormal folding of specific normal cellular proteins (PrP^C^) into the pathological isoform (PrP^Sc^). PrP^Sc^ is protease-resistant, and has a higher proportion of β-sheet structure in place of the normal α-helix structure (Prusiner, 1998; La et al., 2008). Misfolded, aggregated prion proteins lead to brain lesions (Prusiner, 1991). The synthetic neurotoxic prion fragment (PrP106-126) possesses similar physicochemical and pathogenic properties to PrP^Sc^, in that it forms amyloid fibrils with a high β-sheet content, shows partial proteinase K resistance, and is toxic to neuronal cells *in vitro*. Therefore, PrP106-126 is commonly used as a model for the investigation of PrP^Sc^ neurotoxicity (Forloni et al., 1993; Selvaggini et al., 1993; Ettaiche et al., 2000; Gu et al., 2002).

Microglia are the principal immune effector cells in the central nervous system. It has been shown that activated microglial cells are prevalent in regions of the brain with vacuolation, plaque formation, and neuronal damage in individuals with prion diseases (Rezaie and Lantos, 2001). When BV2 microglial cells in culture are exposed to PrP^Sc^, the cells internalize the peptide (Shi et al., 2013). This suggests that microglia may have a similar role in the brain *in vivo*, and they are drawn to prion-rich areas of the brain to participate in the clearance of the damaging proteins. It has also been reported that once microglial clearance of prion proteins is insufficient to remove large aggregates, microglial accumulation at the pathological locus may result in an increase in prion accumulation and inflammatory cytokines release, leading to disease progression (Walter and Neumann, 2009; Hughes et al., 2010).

Microglial activation is thought to play an important role in the pathogenesis of prion diseases through the secretion of pro-inflammatory factors from a specialized complex called the inflammasome. This family of cytosolic molecular platforms are activated by pathogen-associated molecular patterns as well as by endogenous danger signals (Martinon et al., 2006; Mariathasan and Monack, 2007) to trigger innate immune defenses. Within this family, there are many well-known members of different platforms, classified by the different acting domains PYD and HIN (NLR family and PYHIN family). In mammals, the NLR family contains over 20 members, and one of the most studied members is NALP3. Key components of the NALP3 inflammasome are the NALP3 protein (also known as NLRP3), the adaptor protein ASC (apoptosis-associated speck-like protein containing a CARD) and pro-caspase-1 (Schroder and Tschopp, 2010). Inflammasome activation leads to the cleavage of cytokine precursors by caspase-1 and the release of mature pro-inflammatory cytokines such as IL-1β and IL-18 (Mohamed and Dixit, 2009). Our previous studies have demonstrated that the NALP3 inflammasome participates in the activation of microglia induced by neurotoxic prion peptides through the activation of caspase-1 and the subsequent release of active IL-1β (Shi et al., 2012).

Macroautophagy (hereafter referred to as autophagy) is a highly conserved process for the sequestration and degradation of unused or dysfunctional cellular components (e.g., organelles, soluble cytosolic proteins, protein aggregates, and intracellular pathogens) (Listed, 1994; Lin et al., 2013). Autophagy occurs under normal physiological conditions but is especially critical in neurological diseases where pathological protein accumulation occurs, such as Alzheimer’s disease (AD), Parkinson’s disease (PD), Huntington’s disease (HD) and prion diseases (Rubinsztein et al., 2005; Heiseke et al., 2010). Increasing evidence suggests that autophagy is involved in the regulation of immune responses and inflammation that occur in these diseases (Levine et al., 2011). In macrophages treated with autophagy inhibitors or with the deletion of autophagic genes, excessive caspase-1 activation is induced as a result of inflammosome activation, leading to elevated IL-1β secretion in response to LPS stimulation (Saitoh et al., 2008; Kiichi et al., 2011).

Several studies have clearly indicated that protein aggregates, such as those seen in age-related diseases, activate inflammasomes. Activated inflammasomes then provoke low-grade inflammation in tissues (Choi and Ryter, 2011; Levine et al., 2011; Jo et al., 2012), associated with declined autophagic capacity (Salminen et al., 2012). This relationship between inflammasomes and autophagy may also exist in prion diseases.

In this study, we aimed to investigate the relationship between autophagy and PrP106-126-induced NALP3 inflammasome activation in BV2 cells. We showed that the NALP3 inflammasome complex negatively regulates autophagy in response to PrP106-126 stimulation in BV2 microglia. The NALP3 inflammasome attenuated autophagy by activating caspase-1-induced TRIF cleavage. Our findings suggest that inhibiting the activation of caspase-1 may hopefully enhance autophagy, and be an effective therapeutic approach for prion diseases.

## Materials and Methods

### Reagents

Antibodies used in the study included rabbit anti-mouse caspase-1 p10 (sc-514) and anti-mouse ASC (sc-22514-R) (Santa Cruz, CA, United States); rabbit anti-mouse NLRP3 (Abcam, Cambridge, MA, United States); rabbit anti-mouse LC3B (MBL, Japan); and rabbit anti-mouse β-actin (Beyotime, Wuhan, Hubei, China); rabbit anti-rat TLR4 (Proteintech, Chicago, IL, United States); rabbit anti-mouse TRIF (Abcam, Cambridge, MA, United States); rabbit anti-mouse GAPDH (Proteintech, Chicago, IL, United States); goat anti-rabbit secondary antibody and goat anti-mouse secondary antibody (ZSGB Biotechnology, Beijing, China). Lipopolysaccharides (LPS, E. coli L2630) and 3-MA (M9281) were from Sigma-Aldrich (St. Louis, MO, United States), and rapamycin was from Beyotime Biotechnology (Wuhan, Hubei, China). Caspase-1 inhibitor Z-YVAD-FMK was purchased from Biovision (San Francisco, CA, United States). The ELISA kit for mouse IL-1β (88-7013) was purchased from eBioscience Technology (San Diego, CA, United States). Reagents and apparatus used in immunoblotting assays were obtained from Bio-Rad (Hercules, CA, United States).

### Prion Protein Peptide

The PrP peptide PrP106-126 (sequences KTNMKHMAGAAAAGAVVGGLG) was synthesized by Sangon Bio-Tech (Beijing, China). The purity of PrP106-126 was >95% according to data from the synthesizer. The peptide was dissolved in 0.1 M PBS to a concentration of 1 mM, and allowed to aggregate at 37°C for 12 h before incubation with microglial cells. Experiments were conducted with the final peptide concentration of 100 μM and the endotoxin level of working solution was less than 0.05 EU/ml. The endotoxin level of working peptide concentration was measured by the Toxinsensor^TM^ chromogenic limulus amebocyte lysate (LAL) endotoxin assay kit according to the manufacturer’s instructions (GenScript, United States).

### Cell Culture and Treatment Conditions

BV2 cells, a murine microglial cell line, were obtained from the Cell Culture Center Xiehe Medical University (Beijing, China) and cultured in a humidified incubator at 37°C with 5% CO_2_ in RPMI 1640 (Gibco, Grand Island, NY, United States) supplemented with 10% heat-inactivated FBS (Gibco), 100 μg/ml streptomycin, and 100 U/ml penicillin (Gibco).

To induce synthesis of pro-IL-1β, mimicking the chronic activation of microglia in prion disease, BV2 cells were primed with 300 ng/ml LPS for 3 h before treatment with PrP106-126. Priming with LPS was necessary because pro-IL-1β is not constitutively expressed in microglia (Dostert et al., 2008).

For 3-MA (2 mM) (Sigma) and rapamycin (100 nM) (Beyotime Biotechnology) treatment, BV2 cells were primed with 300 ng/ml LPS for 3 h, and were then incubated with PrP106-126 alone, PrP106-126 + 3-MA, or PrP106-126 + rapamycin.

For the treatment inhibiting of caspase-1, BV2 cells were primed with 1 μl/ml of inhibitor Z-YVAD-FMK (Biovision, San Francisco, CA, United States) for 2 h, and were then treated with PrP106-126.

### Small Interfering RNA Transfection

Small interfering (si) RNA were used to silence NLRP3, ASC (Qiagen, Valencia, CA, United State), TLR4, and TRIF (Santa Cruz, CA, United States).

For NLRP3 and ASC siRNA transfection, BV2 cells were plated at 0.8 × 105 cells/well in a 12-well plate and transfected the next day according to the manufacturer’s instructions (HiPerfect Transfection Reagent; Qiagen). Briefly, 75 ng siRNA (siRNA-NLRP3 and siRNA-ASC; Qiagen) were diluted in 100 μl culture medium without serum, and mixed with 3 μl transfection reagent by vortex. The samples were then incubated for 5 to 10 min at room temperature to allow the formation of transfection complexes before adding the complexes onto the BV2 cells. The suppression of NLRP3 and ASC expression by the siRNA was confirmed by western blot analysis.

For TLR4 and TRIF siRNA transfection, BV2 cells were plated at 0.8 × 105 cells/well in a 12-well plate and transfected the next day according to the manufacturer’s instructions (Santa Cruz, CA, United States). On the day of transfection, 3 μl siRNA-TLR4 and 3 μl siRNA transfection reagent (Santa Cruz, CA, United States) were added to 50 μl culture medium without serum, vortexed and then incubated for 30 min at room temperature. Then 400 μl serum-free culture medium was added to the siRNA solution, and incubated again for 12 h at room temperature. The transfection of TRIF siRNA was conducted using the same method except that 4 μl TRIF siRNA was used. TLR4 and TRIF expression in the transfected cells was examined by western blot analysis.

Cell-culture supernatants were assayed for IL-1β by ELISA using a commercial kit (eBioscience Technology) in accordance with the manufacturer’s instructions.

### Extraction of Nuclear and Cytoplasmic Protein and Western Blot Analysis

Cytoplasmic and nuclear proteins of microglial cells were extracted from treated microglia using a protein extraction kit (Cytoplasmic and Nuclear Protein Extraction Kit; Wuhan Boster Biotech). The cellular proteins (40 μg/lane) were separated by SDS-PAGE on 12% gels, and the separated proteins were transferred to nitrocellulose membranes. Non-specific binding sites were blocked by incubating the membrane with 5% fat-free dried milk in Tris-buffered saline (TBS-T, 10 mM Tris, 0.15 M NaCl, 0.05% Tween-20, pH 7.4). The membranes were incubated in rabbit anti-LC3B (1:1000), anti-caspase-1 (1:500), anti-NLRP3 (1:5000), or anti-ASC (1:500) antibodies in TBST at 4°C overnight, washed with TBS-T before incubation with the secondary antibody, either goat anti-mouse IgG or goat anti-rabbit IgG horseradish peroxidase-conjugated antibody (1:5000). Immunoreactive proteins were visualized on an image system (Versadoc, Bio-Rad; Tanon) after incubation with enhanced chemifluorescence (ECF) reagent (170-5060) (Bio-Rad).

### Statistical Analysis

All assays were performed on three separate occasions. Data are expressed as means ± SD. All comparisons for parametric data were made using Student’s test or one way ANOVA followed by *post hoc* Turkey’s test. Non-parametric data (ELISA data) were analyzed by the Kruskal–Wallis ANOVA test followed by the Nemenyi test. SPSS software (version 13.0: SPSS, Inc., Chicago, IL, United States) was used, and *P* < 0.05 was considered significant.

## Results

### Autophagy Negatively Regulates NALP3 Inflammasome Activation in Microglial Cells

**FIGURE 1:**
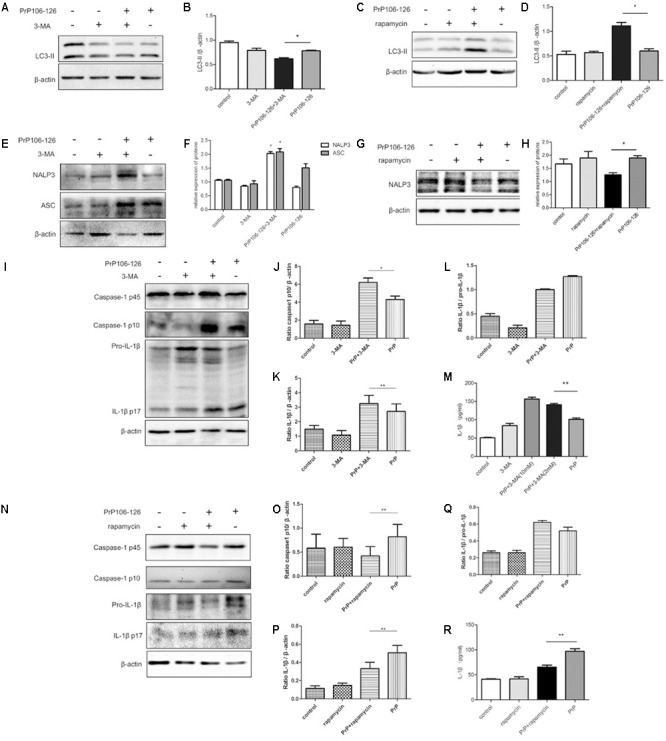
Autophagy negatively regulates NALP3 inflammasome activation in PrP106-126 treated microglial cells. LPS-primed microglial BV2 cells were stimulated with PrP106-126 (100 μM), the autophagy inhibitor, 3-MA (2 mM) and the autophagy inducer, rapamycin (100 nM). **(A,B)** Western blot of LC3-II in lysates of BV2 cells incubated with PrP106-126 and 3-MA for 6 h. **(C,D)** Western blot of LC3 II in lysates of BV2 cells treated with PrP106-126 and rapamycin for 6 h. **(E,F)** Western blot of NALP3 and ASC in lysates of BV2 cells treated with PrP106-126 and 3-MA for 6 h. **(G,H)** Western blot of NALP3 in lysates of LPS-primed BV2 cells treated with PrP106-126 and rapamycin for 6 h. **(I–L)** Western blot analysis of caspase-1, IL-1β, and ratio IL-1β/pro-IL-1βin lysates of BV2 cells treated with PrP106-126 and 3-MA for 12 h. **(M)** ELISA analysis of IL-1β in supernatants of BV2 cells treated with PrP106-126 and 3-MA for 12 h. **(N–Q)** Western blot of caspase-1, IL-1β, and ratio IL-1β/pro-IL-1β in lysates of primed BV2 cells treated with PrP106-126 and rapamycin for 12 h. **(R)** ELISA analysis of IL-1β in supernatants of BV2 cells treated with PrP106-126 and rapamycin for 12 h. Data represent mean ± SD of three separate experiments. ^∗^*P* < 0.05, ^∗∗^*P* < 0.01. Groups which are compared are shown in the graphs.

LC3-II is an autophagy marker and participates in autophagosome formation and maturation (Aoki et al., 2008; He and Klionsky, 2009). We analyzed the expression of LC3-II in LPS-primed BV2 cells in response to PrP106-126 treatment, in the presence and absence of the autophagy inhibitor, 3-MA. Western blot analysis showed that the level of LC3-II was reduced in BV2 cells following treatment with PrP106-126 compared with the untreated control group (**Figures 1A–D**). LC3-II was reduced when the cells were treated with both 3-MA and PrP106-126 (**Figures 1A,B**). As expected, incubation of BV2 cells with both PrP106-126 and the autophagy inducer, rapamycin for 6 h led to an increase in LC3-II protein (**Figures 1C,D**), indicating increased autophagy activity.

Activation of the NALP3 inflammasome assembly is required to trigger caspase-1 cleavage and subsequent IL-1β release (Srinivasula et al., 2002; Sutterwala et al., 2006). Caspase-1 and IL-1β are key inflammatory molecules. Our previous study demonstrated that PrP106-126 induces caspase-1 cleavage and IL-1β release in microglial BV2 cells (Shi et al., 2012). We examined whether PrP-106-126 also induces NALP3 inflammasome activation. The expression of inflammasome proteins, NALP3 and ASC were significantly increased in the peptide-treated BV2 cells pre-incubated with the autophagy inhibitor 3-MA, compared with cells treated with PrP106-126 alone (**Figures 1E,F**). In contrast, the NALP3 protein level was reduced significantly by the autophagy inducer, rapamycin, suggesting that enhanced autophagy suppress the expression of NALP3 protein (**Figures 1G,H**).

We next examined whether treatment with 3-MA affected the activation of caspase-1 in PrP106-126-treated BV2 cells. First we confirmed that PrP106-126 induced an increase in cleaved, bioactive caspase-1 (p10) in BV2 cells by repeating our previous experiment (Shi et al., 2012). Then following co-treatment with 3-MA and PrP106-126, we observed a marked increase in the expression of caspase-1 p10 in cell lysate (**Figures 1I,J**). Similarly, mature IL-1β was in greater abundance in both cell lysate and culture medium of BV2 cells treated with 3-MA and PrP106-126 than in cells treated with PrP106-126 alone (**Figures 1K–M**), indicating that the autophagy inhibitor promoted the conversion of pro-IL-1β to its bioactive form, IL-1β p17. At the highest test concentration (10 mM), 3-MA strongly induced IL-1β secretion. Collectively these results demonstrated that inhibition of autophagy in BV2 microglia enhances neurotoxic prion peptide-induced caspase-1 activation and IL-1β activation and secretion. To confirm that the enhanced caspase-1 activation and IL-1β secretion described above were due to autophagy inhibition, we investigated whether we could achieve the opposite results by stimulating autophagy with the autophagy inducer, rapamycin. In contrast to the finding with 3-MA, rapamycin inhibited PrP106-126-induced activation of caspase-1 (p45) (**Figures 1N,O**), and microglial cells produced decreased level of caspase-1 p10. In addition, rapamycin also inhibited the expression of pro-IL-1β in cell lysate and the release of mature IL-1β into the cell culture medium after treatment with PrP106-126 (**Figures 1P–R**).

Taken together, the opposite results obtained by the pharmacologic inhibition and activation of autophagy suggest that autophagy negatively regulate inflammasome complex formation, activation of caspase-1 and subsequent release of the pro-inflammatory cytokine IL-1β.

### Gene Silencing of NALP3 Inflammasome Components Augments Autophagy

The above results demonstrate that the inhibition of autophagy aggravates inflammasome activity and subsequent release of pro-inflammatory cytokines. We hypothesized that inflammasome activation could negatively regulate autophagy in PrP106-126-induced microglial activation.

**FIGURE 2:**
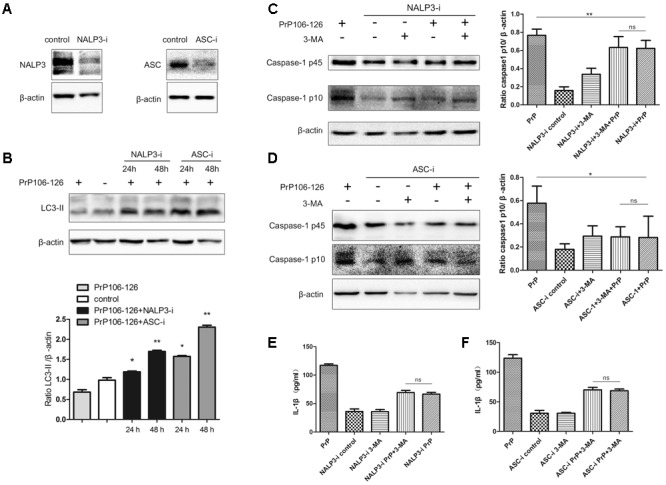
Gene silencing of NALP3 inflammasome components augments autophagy. **(A)** Western blot analysis of NALP3 and ASC in lysates of NALP3 or ASC siRNA-transfected BV2 microglia cells. **(B)** Western blot analysis of LC3-II in lysates of NALP3 or ASC siRNA-transfected BV2 microglia cells with exposure to PrP106-126. **(C,D)** Western blot analysis of caspase-1 in lysates of NALP3 or ASC siRNA-transfected BV2 cells treated with PrP106-126 and 3-MA (2 nM) for 12 h. **(E,F)** ELISA analysis of IL-1β in supernatants of primed- and NALP3 or ASC siRNA-transfected BV2 cells treated with PrP106-126 and 3-MA (2 nM) for 12 h. Data represent mean ± SD of three separate experiments. ^∗^*P* < 0.05, ^∗∗^*P* < 0.01. Groups which are compared are shown in the graphs.

To confirm our hypothesis, we prepared NALP3 and ASC-knockdown BV2 cells using siRNA. The efficiency of siRNA-mediated knockdown of NALP3 and ASC was evaluated 48 h after siRNA transfection by western blot analysis. Expression of both proteins was significantly reduced at the protein level, NALP3 by 57% and ASC by 53% (**Figure 2A**). We then assessed LC3-II in the cell lysate as a measure of autophagy. LC3-II expression was significantly enhanced in NALP3- and ASC-knockdown BV2 cells incubated with PrP106-126 (**Figure 2B**), suggesting that suppression of inflammasome enhances autophagy. The co-incubation of BV2 cells with PrP106-126 and autophagy inhibitor 3-MA (2 nM) resulted in the up-regulation of cleaved caspase-1 p10 and release of IL-1β. At this part, it was shown that knockdown of NALP3 or ASC partially blocked the up-regulation of cleaved caspase-1 p10 by 3-MA and PrP106-126 (**Figures 2C,D**). Similarly, the bioactive IL-1β in the above-mentioned condition was also down-regulated by knockdown of NALP3 inflammasome components (**Figures 2E,F**). These findings indicate that gene silencing of NALP3 inflammasome components rescue down-regulated autophagy.

### PrP106-126-Induced Autophagy Is Partly Mediated by the TLR4-TRIF Pathway

**FIGURE 3:**
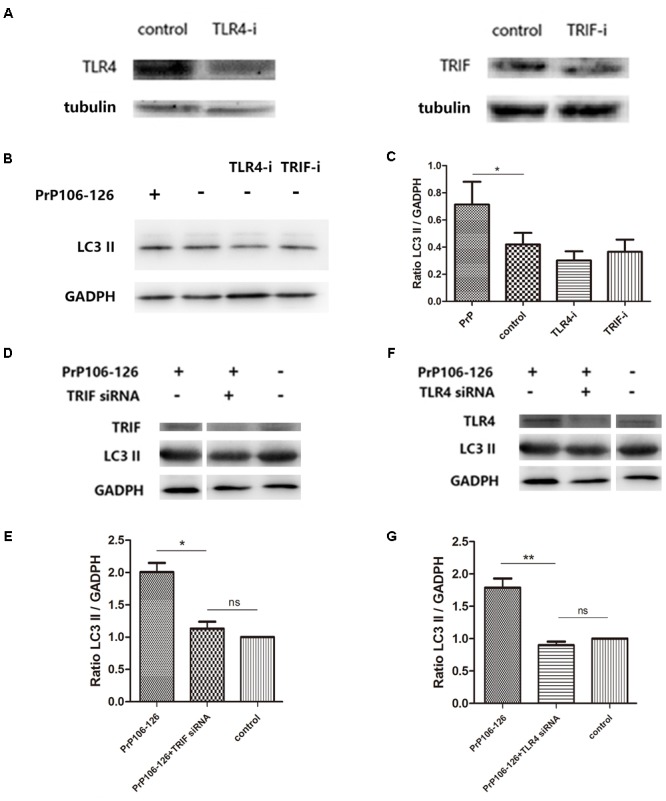
TLR4 or TRIF knockdown suppresses PrP106-126-induced autophagy. **(A)** Western blot analysis of TLR4 and TRIF in lysates of TLR4 and TRIF siRNA-transfected BV2 microglia. **(B,C)** Western blot analysis of LC3-II in lysates of TLR4 or TRIF siRNA-transfected without PrP106-126. **(D,E)** Western blot analysis of LC3-II in lysates of TRIF siRNA-transfected BV2 cells exposed to PrP106-126 for 12 h. **(F,G)** Western blot analysis of LC3-II in lysates of TLR4 siRNA-transfected BV2 cells exposed to PrP106-126 for 12 h. Data represent mean ± SD of three separate experiments. ^∗^*P* < 0.05. Groups which are compared are shown in the graphs. ^∗∗^*P* < 0.01.

It has been shown that autophagy activation in response to *Pseudomonas aeruginosa* infection is mediated via TLR4 and TRIF (Jabir et al., 2014). We hypothesized that this pathway is also involved in PrP106-126-induced autophagy in BV2 cells. To prove this, we knocked down TLR4 and TRIF by siRNA and assessed PrP106-126-induced autophagy. The efficiency of TLR4 and TRIF knockdown was evaluated 48 h after siRNA transfection by western blot analysis. Expression of both proteins was significantly diminished, TLR4 by 57% and TRIF by 50% (**Figure 3A** and **Supplementary Figure S1**). Expression of LC3-II, which is an autophagy marker, was not significant influenced by siRNA transfection (**Figures 3B,C** and **Supplementary Figure S1**), but was decreased by 50% in lysates of TLR4- and TRIF-knockdown BV2 cells exposed to PrP106-126, compared with the positive control (untransfected cells exposed to PrP106-126) (**Figures 3D–G** and **Supplementary Figure S1**). These findings suggest that the TLR4-TRIF pathway plays a significant role in autophagy induced by PrP106-126.

### Caspase-1 Induces TRIF-Cleavage

**FIGURE 4:**
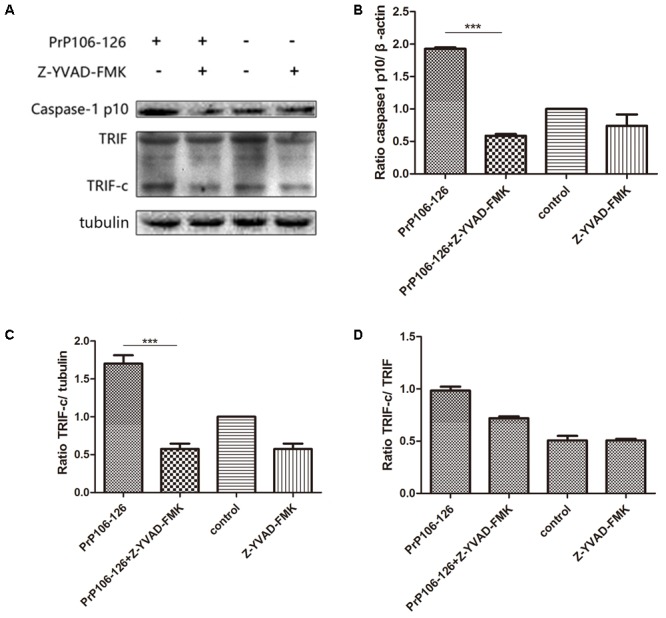
Prion peptide-induced TRIF cleavage is mediated by activated caspase-1. **(A)** Western blot analysis of cleaved-TRIF in lysates from BV2 cells treated with PrP106-126 and the caspase-1 inhibitor, Z-YVAD-FMK. BV2 cells were incubated with the caspase-1 inhibitor for 2 h followed by PrP106-126 (100 μM) for an additional 12 h. **(B–D)** Densitometric measurement of caspase-1 (p10) relative to β-actin, TRIF-c relative to tubulin and, TRIF-c to full-length TRIF ratio. Data represent mean ± SD of three separate experiments. ^∗∗∗^*P* < 0.001. Groups which are compared are shown in the graphs.

As described above, both NALP3 inflammasome and autophagy influence caspase-1 activation. We investigated whether caspase-1 as a protease cleaves TRIF, which was shown above as an important factor in autophagy activation. As expected, addition of PrP106-126 resulted in activation of caspase-1 in BV2 cells (**Figures 4A,B** and **Supplementary Figure S2**). PrP106-126 also stimulated cleavage of TRIF (**Figures 4A,C, Supplementary Figure S2**). Then we showed that pre-incubation of microglial cells with the caspase-1 inhibitor, Z-YVAD-FMK reduced the cleaved-TRIF level induced by PrP106-126 (**Figure 4C** and **Supplementary Figure S2**). When compared to the full-length TRIF, TRIF-c expression also declined significantly in the group treated with the caspase-1 inhibitor (**Figure 4D** and **Supplementary Figure S2**). These results suggest that caspase-1 induces the cleavage of TRIF.

### Caspase-1-Mediated TRIF Cleavage Regulates PrP106-126-Induced Autophagy

**FIGURE 5:**
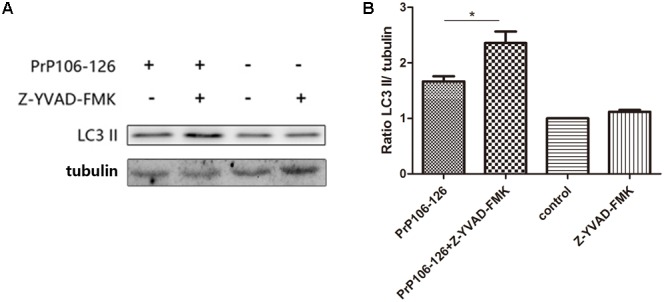
Prevention of caspase-1-induced TRIF cleavage augments autophagy. **(A)** Western blot analysis of cleaved-TRIF in lysates of BV2 cells. Primed cells were either left unstimulated, or stimulated with PrP106-126 alone, or stimulated with caspase-1 inhibitor alone, or stimulated with caspase-1 inhibitor for 2 h followed by PrP106-126 (100 μM) for an additional 12 h. **(B)** Densitometric measurement of the ratio of LC3 II to β-tublin. Data represent the mean ± SD and are representative of three separate experiments. ^∗^*P* < 0.05, significantly different from control.

To confirm the influence of caspase-1 activation on autophagy, we assessed the autophagy level in microglial cells pre-incubated with the caspase-1 inhibitor, Z-YVAD-FMK before incubation with PrP106-126. Pre-incubation of microglial cells with the caspase-1 inhibitor significantly increased prion peptide-induced LC3 II expression (by 1.5 times) compared with the un-pretreated cells (**Figures 5A,B** and **Supplementary Figure S2**). These findings suggest that suppression of caspase-1 activation enhanced peptide-induced autophagy probably as result of suppressed TRIF cleavage by caspase-1.

## Discussion

Earlier studies link autophagy to innate immunity as a conserved host defense response against diverse intracellular pathogens (Sumpter and Levine, 2010; Deretic et al., 2013; Faure and Lafont, 2013; Teh and Hofius, 2014). The regulation of autophagy during infection is complex, and mediated by numerous receptors, including Toll-like receptors (TLRs) and members of the Nod-like receptor (NLR) family (Bortoluci and Medzhitov, 2010; Xu et al., 2013). Downregulation of autophagy in mice by deletion of the ATG16L1 gene, which is essential for autophagy, resulted in inflammasome activation and increased severity of experimental colitis (Rubinsztein et al., 2005). We were interested in exploring the possibility that the opposite might be true, i.e., the activation of autophagy might lead to a decrease in the inflammatory response, in an *in vitro* prion model. Through both the inhibition and induction of autophagy in BV2 microglial cells exposed to the prion peptide PrP106-126, we showed that autophagy negatively regulates the NLRP3 inflammasome activity. Incubation of microglial cells with the autophagy inhibitor, 3-MA enhanced inflammasome-dependent activation of caspase-1 and the subsequent release of IL-1β, while the autophage inducer, rapamycin had the opposite effect.

IL-1β is an important pro-inflammatory cytokine that is processed into its mature form from its inactive precursor by caspase-1 (Peyrin et al., 1999). We showed that in microglial cells, induction of autophagy by rapamycin attenuated the expression of pro-IL-1β and mature IL-1β release. This is consistent with other studies that have showed that autophagy can suppress inflammatory reactions (Saitoh et al., 2008; Jo et al., 2012). LPS stimulation of macrophages triggered autophagy, which leads to IL-1β being sequestered into autophagosomes, and induction of autophagy by rapamycin resulted in the degradation of intracellular pro-IL-1β and inhibited secretion of mature IL-1β (Harris et al., 2011). Conversely depletion of autophagy proteins enhanced the conversion of pro-IL-1β to its active form as a result of caspase-1 activation (Kiichi et al., 2011). Collectively the above studies suggest that autophagy controls inflammation by the degradation of pro-IL-1β. Stimulation of autophagy could be an effective tool for controlling inflammation in prion diseases.

The TLR adaptor, TRIF has been shown to be an important factor in the induction of NLRP3 inflammasome activation (Rathinam et al., 2012). In different disease conditions, the pathway of NLRP3 inflammasome activation may differ, and could be TLR4-MyD88 (Calil et al., 2014), TLR4-TRIF (Jabir et al., 2014), or both pathways (Yan et al., 2017). A study in the *Pseudomonas aeruginosa* infection model showed that caspase-1 directly cleaved TRIF, resulting in inhibition of TLR4-associated signaling and autophagy, as well as NLRP3 inflammasome activation (Jabir et al., 2014). Autophagocytic responses to bacterial infection limit inflammasome activation and resultant IL-1β secretion. We also demonstrated that suppression of TRIF or TLR4 by siRNA diminished PrP106-126-induced autophagy in microglial cells, while the inhibition of caspase-1 blocked TRIF cleavage and enhanced autophagy, indicating that the TLR4-TRIF signaling pathway was involved in PrP106-26-induced autophagy. These findings suggest that caspase-1-mediated TRIF cleavage is a molecular mechanism for the negative interactions between autophagy and inflammasome activation. NLRP3 inflammasome negatively controls autophagy partly by activating caspase-1-mediated TRIF cleavage.

In a recently published work, Nuvolone et al. (2014) demonstrated that NALP3 and ASC have limited role in prion pathogenesis *in vivo* model with RML6 strain. This might be due to strain dependent differences in prion infections. Strain-dependent variation in prion infection has been previously studied by many research groups (Philippe et al., 2010; Ayers et al., 2011). The strain-dependent differences was also observed in human prion diseases (Baker et al., 1999; Ayers et al., 2011). We previously shown that prion peptide PrP106-126 induced neurotoxic insults involved active participation of NALP3 inflammasomes (Shi et al., 2012, 2013), similarly, Iva and coworkers shown the involvement of NALP3 inflammasomes in α-PrP fibrils induced toxicity (Hafner-Bratkovic et al., 2012). Therefore, we have to bear in mind when we search for a cure in prion disease, that different prion particles can lead to different cell activations.

## Conclusion

**FIGURE 6:**
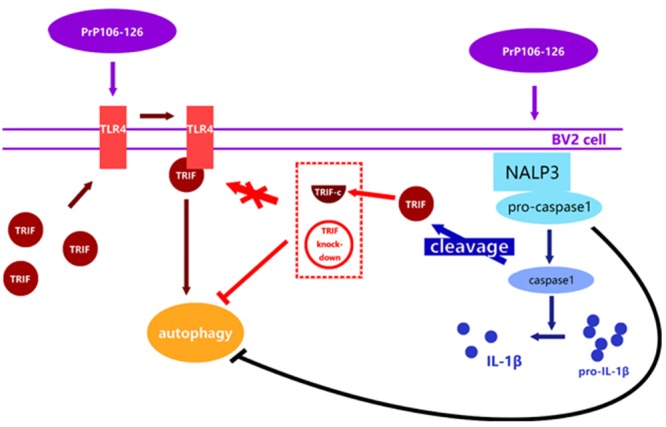
Proposed model of negative regulation of autophagy by NALP3 imflammasome. Autophagy acts to downregulate NLRP3 inflammasome activation and subsequent caspase-1 cleavage and IL-1β release in PrP106-126-treated microglia, while the NLPR3 inflammasome limits autophagy cleaving TRIF via activated caspase-1.

This study is the first attempt to investigate the reciprocal regulation between inflammasome and autophagy in prion diseases. We observed that autophagy acts to downregulate NLRP3 inflammasome activation and subsequent caspase-1 cleavage and IL-1β release in PrP106-126-treated microglia, while the NLPR3 inflammsome limits autophagy via activated caspase-1 cleaving TRIF (**Figure 6**). Chronic inflammation is a common feature of numerous neurodegenerative diseases. In prion diseases, autophagy may play a cytoprotective role by aiding in the digestion of misfolded aggregates (Dziedzic and Caplan, 2012). Upregulation of autophagy by inhibiting the activation of caspase-1 may present a novel therapeutic approach to the treatment of prion diseases, simultaneously leading to a reduction of neuroinflammation and accelerating the removal of misfolded proteins.

## Author Contributions

All authors listed have made a substantial, direct and intellectual contribution to the work, and approved it for publication.

## Conflict of Interest Statement

The authors declare that the research was conducted in the absence of any commercial or financial relationships that could be construed as a potential conflict of interest. The reviewer AG-Q and handling Editor declared their shared affiliation.

## Supplementary Material

FIGURE S1 The uncut membranes photos for **Figure 3**.

FIGURE S2 The uncut membranes photos for **Figures 4, 5**.
