# Supplementary figures and images for: The NLRP3-Caspase 1 Inflammasome Negatively Regulates Autophagy via TLR4-TRIF in Prion Peptide-Infected Microglia
Source: Front Aging Neurosci. 2018 Apr 18;10:116. doi: 10.3389/fnagi.2018.00116 (PMC5915529; doi:10.3389/fnagi.2018.00116)

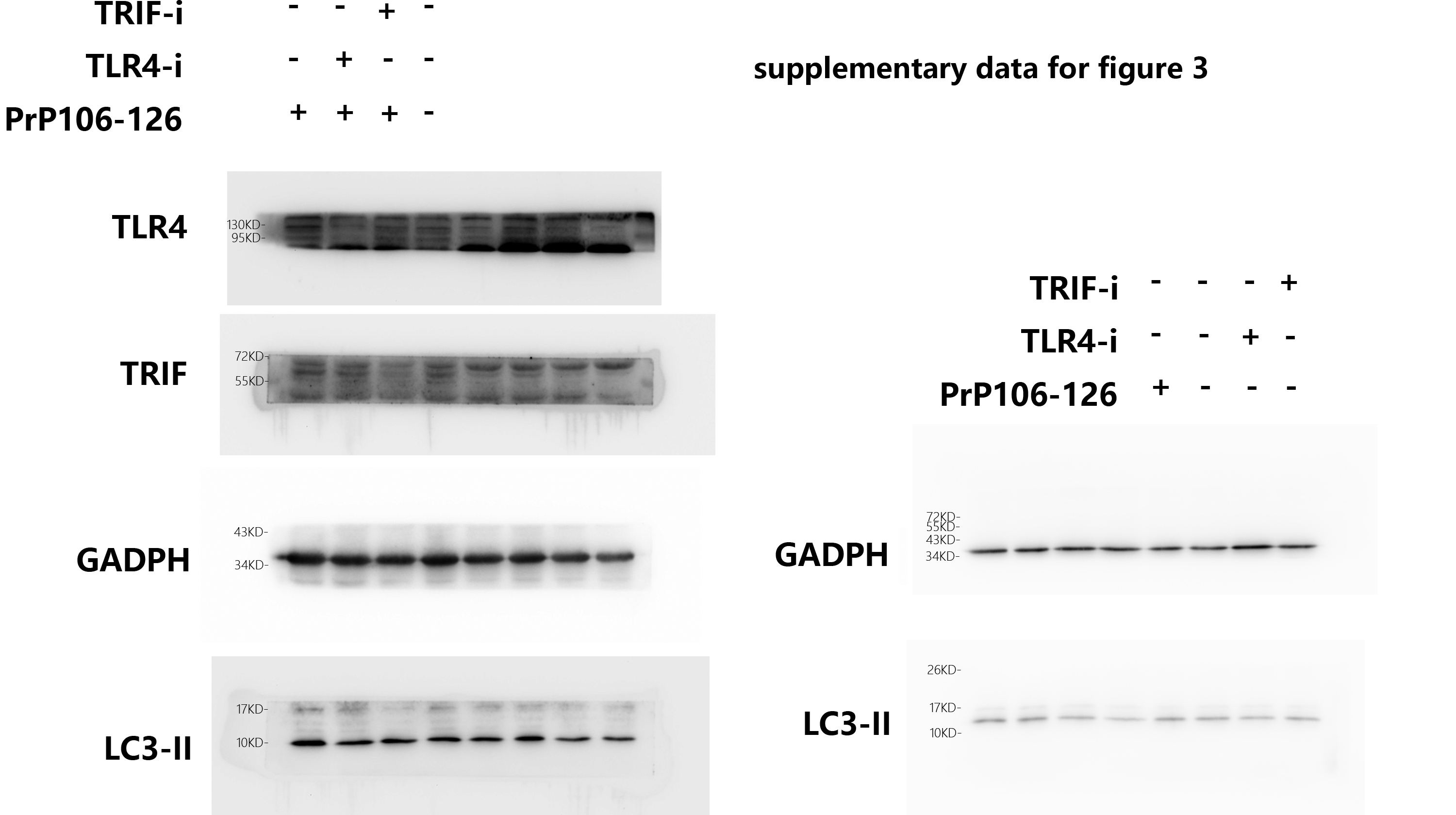

Supplement: FIGURE S1 — The uncut membranes photos for Figure 3. [file Image_1.JPEG]

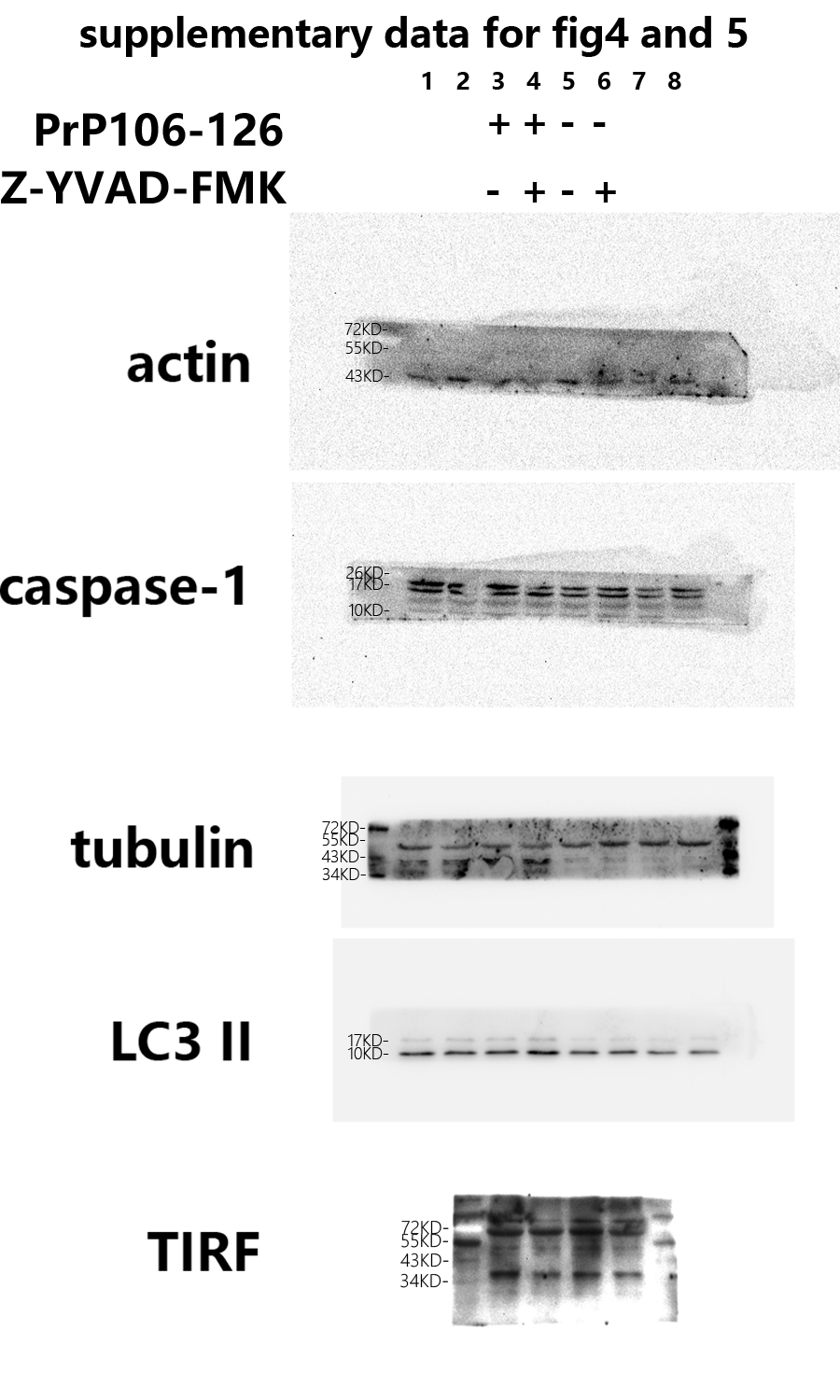

Supplement: FIGURE S2 — The uncut membranes photos for Figures 4, 5. [file Image_2.JPEG]
